# Antiosteoporosis effect of bryodulcosigenin on ovariectomy-induced osteoporosis in experimental rats

**DOI:** 10.1590/acb391024

**Published:** 2024-04-22

**Authors:** Kai Sun, Lin Qin

**Affiliations:** 1 Yunnan University – The Affiliated Hospital – Department of Spinal Surgery – Kunming, China.; 2 Kunming Medical University – School of Pharmaceutical Science and Yunnan Key – Laboratory of Pharmacology for Natural Products – Kunming, Yunnan, China.; 3 Kunming Medical University – The First Affiliated Hospital – Department of Endocrinology – Kunming, China.

**Keywords:** Bone diseases, Osteoporosis, Inflammation, Hormones

## Abstract

**Purpose:**

Osteoporosis is a bone disease which commonly occurred in postmenopausal women. Almost 10 percent of world population and approximately 30% of women (postmenopausal) suffer from this disease. Alternative medicine has great success in the treatment of osteoporosis disease. Bryodulcosigenin, a potent phytoconstituent, already displayed the anti-inflammatory and antioxidant effect. In this study, we made effort to analyze the antiosteoporosis effect of bryodulcosigenin against ovariectomy (OVX) induced osteoporosis in rats.

**Methods:**

Swiss albino Wistar rats were grouped into fIve groups and given an oral dose of bryodulcosigenin (10, 20 and 30 mg/kg) for eight weeks. Body weight, uterus, bone mineral density, cytokines, hormones parameters, transforming growth factor (TGF)-β, insulin-like growth factor (IGF), osteoprotegerin (OPG), receptor activator of nuclear factor kappa-Β ligand (RANKL), and its ratio were estimated.

**Results:**

Bryodulcosigenin significantly (p < 0.001) suppressed the body weight and enhanced the uterine weight and significantly (p < 0.001) increased the bone mineral density in whole femur, caput femoris, distal femur and proximal femur. Bryodulcosigenin significantly (P < 0.001) altered the level of biochemical parameters at dose dependent manner, significantly (P < 0.001) improved the level of estrogen and suppressed the level of follicle stimulating hormone and luteinizing hormone. Bryodulcosigenin significantly (P < 0.001) improved the level of OPG and suppressed the level of RANKL.

**Conclusions:**

Bryodulcosigenin reduced the cytokines level and suppressed the TGF-β and IGF. We concluded that bryodulcosigenin is an antiosteoporosis medication based on the findings.

## Introduction

Osteoporosis is a condition in which the bones become weak and brittle, making them more likely to break. The condition is caused by a loss of bone density, which results in a disruption of the micro-architecture of the bone tissue. This can lead to an increased risk of fractures, particularly in the hips, spine, and wrists. Risk factors for osteoporosis include being female, advancing age, low body weight, smoking, and lack of physical activity. Treatment for osteoporosis includes medications to slow bone loss and increase bone density, as well as lifestyle changes such as regular exercise and a diet rich in calcium and vitamin D^1^^,^ 2. It is a common disease identify worldwide, approximately 10% of world population and around 30% of postmenopausal women suffer from this disease^3^. The main reason for the development of the osteoporosis is poor life quality, and the disease increases the aging population cases. As a result, osteoporosis has a large socioeconomic impact, as well as augmented mortality and fractures^4^. Menopause is the most common condition in women, and it is connected to an increased risk of osteoporosis. This is caused by an imbalance between bone resorption capacity and osteoclast development due to hormone loss or changes, particularly estrogen (E_2_)^5^. The reduction level of E_2_ hormones act as an effective inhibitor of bone density loss and ensuring the induction of osteoporosis^1^^,^ 2. 

Osteoporosis is a condition characterized by a decrease in bone mineral density (BMD), which can lead to an increased risk of fractures. This decrease in BMD can be caused by alterations in hormones, such as estrogen deficiency in postmenopausal women, and can result in dysfunction of cancellous (spongy) bone in the metaphysis (the area near the growth plate) of long bones^6^^,^ 7. Osteoclasts and osteoblasts are the two main types of cell involved in bone remodeling. Osteoclasts are responsible for breaking down and reabsorbing old bone, while osteoblasts are responsible for building new bone. Together, these two cell types work to maintain the balance of bone metabolism and integrity^8^^,^ 9. Osteoprotegerin (OPG) is a protein that acts as a decoy receptor for receptor activator of nuclear factor kappa-Β ligand (RANKL), binding to it and preventing it from activating osteoclasts. This helps to regulate the balance between osteoclasts and osteoblasts and prevent excessive bone resorption. RANKL, on the other hand, is a protein that binds to and activates the RANK on the surface of osteoclasts, promoting the formation and activation of these cells. RANKL is produced by osteoblasts and stromal cells and is involved in the regulation of bone remodeling. Dysregulation of OPG and RANKL signaling can lead to bone disorders such as osteoporosis, osteoarthritis, and other bone metabolic diseases. The balance between RANKL and OPG is critical for the maintenance of bone homeostasis, and the drugs are being developed targeting these molecules to treat the bone disorders^10^^,^ 11.

Therapeutic treatment available for the osteoporosis are hormone therapy, mineral supplement, vitamin, along with the antiosteoporosis drugs^12^^,^ 13. Menopause is regulated via hormone replacement therapy (HRT), but HRT has limitations due to various serious side effects, like promotion and induction of breast, endometrial, and ovarian cancer^14^. Therefore, effective strategies for the treatment of osteoporosis such as physical activity and proper nutrients diets might decrease the risk^15^^,^ 16. However, this treatment is not effective for osteoporosis. Nowadays, the focus of the investigations is a more effective alternative system of medicine on osteoporosis with less side effects1^7^^,^ 18. Furthermore, more research to reduce the osteoporotic bone loss is needed.

Bryodulcosigenin is a naturally occurring cucurbitane-type triterpenoid compound found in plants^19^. Cucurbitane-type triterpenoids are a class of compounds that are commonly found in members of the Cucurbitaceae family, which includes plants such as gourds, cucumbers, and melons^19^^–^ 21. These compounds have been found to have various biological activities, such as antioxidant, anti-inflammatory, and anticancer properties^19^^,^ 20 ^,^ 22.

## Methods

### Chemical

Bone-specific alkaline phosphatase (bALP), tartrate-resistant acid phosphatase (TRAP), osteoprotegerin, osteocalcin, and Cterminal telopeptide of type I collagen (CTX) were measured using commercial kits according to the manufacturer’s instructions (Cobas Roche, Basel, Switzerland). Tumor necrosis factor-α (TNF-α), interleukin-6 (IL-6), interleukin-1β (IL-1β), insulin-like growth factor (IGF), interferon-gamma (IFN-γ), and transforming growth factor (TGF)-β were determined using the commercially available kits following the manufacture procedure (Jiancheng Bio, Nanjing, China).

### Animals

Swiss albino rats (aged: 10–12 weeks, weighing 220–250 g, sex: female) were used in current study. The rats were kept in the standard laboratory conditions (temperature 20 ± 5°C; relative humidity 65% with 12 h dark and light cycle). The entire experiment followed the Guide for the Care and Use of Laboratory Animals. During the whole experimental study, the care was taken to minimize the distress, pain, and discomfort to the animal.

### Induction of ovariectomy

For the induction of osteoporosis, previously reported method was used5. The rats were anesthetized using the intraperitoneal injection of pentobarbitone (50 mg/kg, b.w.) and subjected to the ovariectomy. Briefly, the dorsal muscles and rat skin were cut, and fat tissue around the ovary was removed. The junction between the fallopian tube and uterine horn was clamped, and small cut was made to remove the ovary. For the normal control, the same procedure was carried out, but the ovary tissue was not exposed.

### Experimental protocol

The rats acclimatized for one week in the laboratory for adopting the condition. After the acclimatization period, the rats were divided into different groups, as follows:

- Group A: normal control;
- Group B: OVX control (received vehicle only);
- Group C: OVX + bryodulcosigenin (10 mg/kg);
- Group D: OVX + bryodulcosigenin (20 mg/kg);
- Group E: OVX + bryodulcosigenin (30 mg/kg).

The rats were given the treatment described before for eight weeks. All the rats’ body weights were measured at regular intervals. The rats were fasted overnight before the end of the experiment, and blood samples were taken from the retro orbital plexus under anesthesia. To separate the serum samples, the blood samples were centrifuged. For subsequent biochemical parameter measurement, the separated serum samples were stored at -20°C.

### Organ separation

The vagina and uterine tissue were separated out and weighted. The absolute weight of uterine and vagina was estimated using the following formula.

### Bone mineral density estimation

Thiopental sodium was used for anesthetized the rats, and right femurs of all group rats were used for the estimation of BMD using the dual energy X ray absorptiometry machine (Lunar iDXA, USA). All analyzed were collected by cropping the rats with their knees flexed and their hips stretched. The scans were all evaluated and saved.

### Bone turnover

The bone turnover markers TRAP, osteoprotegerin, CTX, bALP, and osteocalcin were measured using commercial kits according to the manufacturer’s instructions (Cobas Roche, Basel, Switzerland).

### Urine calcium, serum calcium and phosphorus

Using a flame photometer, the previous published method for estimating calcium levels in urine was employed (EHST, China)^3^. The phosphorus and calcium were estimated in the serum using the arsenazo-3 dye and ammonium molybdate colorimetric procedure.

### Hormone

The hormone such as LH, FSH, and E2 were estimated using the radioimmunoassay method (Subbio, Inc., China). The level of oxytocin (OT) was estimated via following the manufacture instruction (WKEA Med. supplies, Changchun, China) assays.

### Plasma enzyme

Previously reported method was used for the determination of the level of plasma enzyme like tartrate-resistant acid phosphatase (TRAP) and bone alkaline phosphatase (ALP) with minor modification^23^^,^ 24.

### Cytokines

Cytokines such as TNF-α, IL-6, IL-1β, IGF, IFN-γ, and TGF-β were determined using the commercially available kits following the manufacture procedure (Jiancheng Bio, Nanjing, China).

### OPN and receptor activator of nuclear factor kappa-B ligand

The levels of OPN and RANKL were estimated using the commercially available kits (Jiancheng Bio, Nanjing, China) following the manufacture instruction.

### Statistical analysis

The statistical analysis was performed using GraphPad Prism software (St. Louis, USA). Scanning electron microscope was used to exhibit all the data. The Dunnett’s test was used to compare all the groups, and P < 0.05 was considered statistically significant.

## Results

### Body and organ weight

**Figure 1:**
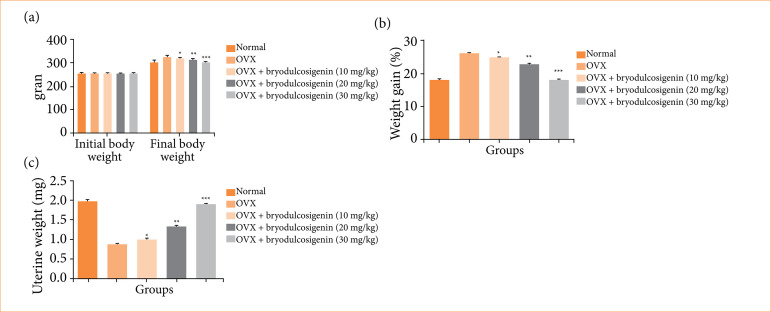
The effect of bryodulcosigenin on the body weight and organ weight OVX induced osteoporosis in rats. **(a)** Body weight; **(b)** weight gain; **(c)** uterine weight.

In all groups, we selected the similar weight of rats. The OVX-induced group rats had a higher body weight once the experiment was completed. Bryodulcosigenin treatment significantly (P < 0.001) declined the body weight as dose dependent (Fig. 1a). Figure 1b exhibited the percentage improvement of body weight in the OVX group rats. The normal group rats improved their body weight percentage in the expected manner, whereas the OVX-induced group rats improved their body weight the most.

OVX: ovariectomy; *P < 0.05, **P < 0.01 and ***P < 0.001 were considered as significant.

The uterine weight of different groups of rats is shown in Fig. 1c. On the comparison between the groups, OVX induced group showed reduction in the uterine weight, and bryodulcosigenin remarkably enhanced the body weight.

### Bone mineral density

**Figure 2:**
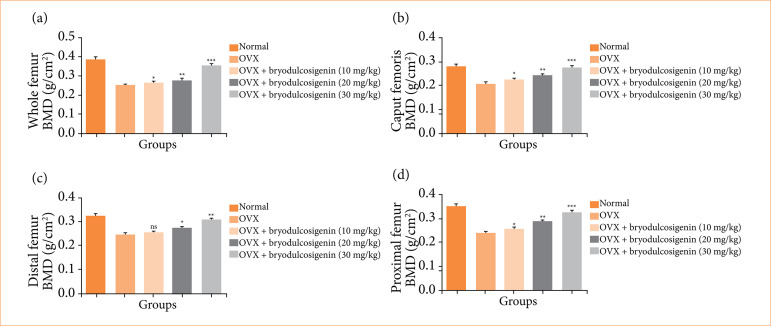
The effect of bryodulcosigenin on the bone mineral density level against OVX induced osteoporosis in rats. **(a)** Whole femur, **(b)** caput femoris, **(c)** distal femur; **(d)** proximal femur.

We determined the BMD level in whole femur (Fig. 2a), caput femoris (Fig. 2b), distal femur (Fig. 2c), and proximal femur (Fig. 2d). The total femur, caput femoris, distal femur, and proximal femur of OVX-induced group rats had decreased BMD, and bryodulcosigenin treatment significantly (P < 0.01) reversed the level of BMD.

OVX: ovariectomy; *P < 0.05, **P < 0.01 and ***P < 0.001 were considered as significant.

### Biochemical parameters

**Figure 3:**
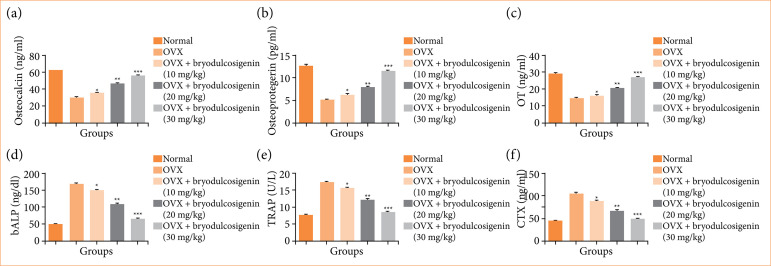
The effect of bryodulcosigenin on the biochemical parameters against OVX induced osteoporosis in rats. **(a)** Osteocalcin, **(b)** osteoprotegerin, **(c)** OT, **(d)** bALP, **(e)** TRAP; **(f)** CTX.

OVX: ovariectomy; OT: oxytocin; bALP: bone-specific alkaline phosphatase; TRAP: tartrate-resistant acid phosphatase; CTX: Cterminal telopeptide of type I collagen; *P < 0.05, **P < 0.01 and ***P < 0.001 were considered as significant.

When compared to normal group rats, OVX induced group rats had lower levels of osteocalcin (Fig. 3a), osteoprotegerin (Fig. 3b), OT (Fig. 3c), and higher levels of bALP (Fig. 3d), TRAP (Fig. 3e), and CTX (Fig. 3f). Bryodulcosigenin remarkably increased the levels of osteocalcin, osteoprotegerin, and OT, while suppressed the levels of bALP, TRAP, and CTX.

### Hormones level

**Figure 4:**
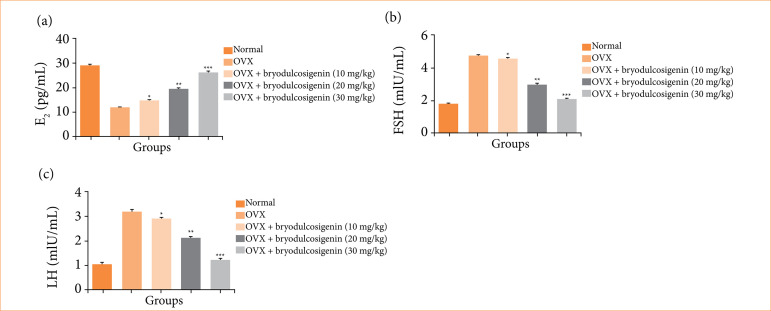
The effect of bryodulcosigenin on the hormone level against OVX induced osteoporosis in rats. **(a)** E2, **(b)** FSH **(c)** LH.

OVX-induced group rats had lower estrogen levels (Fig. 4a) and higher level of FSH (Fig. 4b), and LH levels (Fig. 4c), and bryodulcosigenin treatment significantly (P < 0.001) restored hormone levels in a dose-dependent manner.

OVX: ovariectomy; *P < 0.05, **P < 0.01 and ***P < 0.001 were considered as significant.

Osteoprotegerin, receptor activator of nuclear factor kappa-B ligand and osteoprotegerin/receptor activator of nuclear factor kappa-B ligand ratio

**Figure 5:**
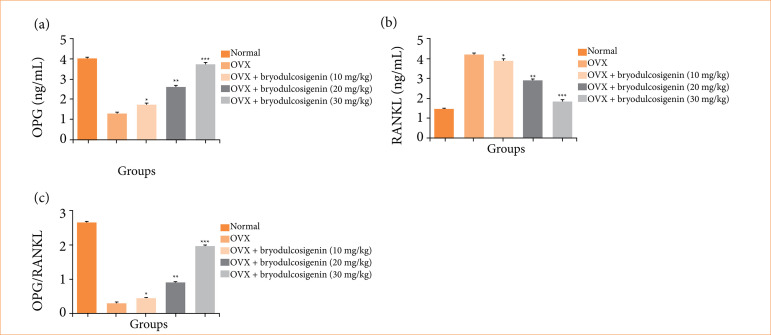
The effect of bryodulcosigenin on the OPG, RANKL and OPG/RANKL ratio against OVX induced osteoporosis in rats. **(a)** OPG, **(b)** RANKL, **(c)** OPG/RANKL ratio.

OVX: ovariectomy; OPG: osteoprotegerin; RANKL: receptor activator of nuclear factor kappa-Β ligand; *P < 0.05, **P < 0.01 and ***P < 0.001 were considered as significant.

The OVX-induced group rats had lower levels of OPG, OPG/RANKL ratio, and higher levels of RANKL. Treatment with bryodulcosigenin significantly (P < 0.001) increased the OPG level, and OPG/RANKL ratio and reduced the RANKL level (Fig. 5).

Transforming growth factor-*β* and insulin-like growth factor

**Figure 6:**
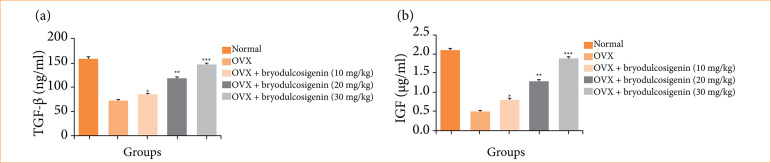
The effect of bryodulcosigenin on the TGF-β and IGF against OVX induced osteoporosis in rats. **(a)** TGF-β, **(b)** IGF.

OVX: ovariectomy; TGF: transforming growth factor; IGF: insulin-like growth factor; *P < 0.05, **P < 0.01 and ***P < 0.001 were considered as significant.

When compared to normal group rats, OVX induced group rats had lower levels of TGF-β (Fig. 6a) and IGF (Fig. 6b). Treatment with bryodulcosigenin (P < 0.001) increased TGF-β and IGF levels in a dose-dependent manner.

### Cytokines

**Figure 7:**
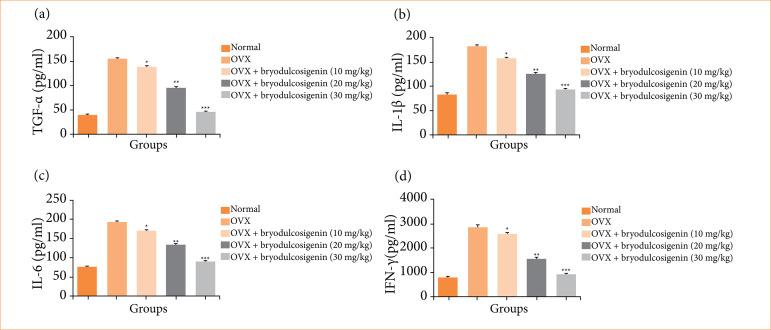
The effect of bryodulcosigenin on the cytokines level against OVX induced osteoporosis in rats. **(a)** TNF-α, **(b)** IL-1β, **(c)** IL-6, **(d)** IFN-γ.

OVX: ovariectomy; TNF-α: tumor necrosis factor-α; IL-1β: interleukin-1β; IL-6: interleukin-6; IFN-γ: interferon-gamma; *P < 0.05, **P < 0.01 and ***P < 0.001 were considered as significant.

The effect of bryodulcosigenin on the normal and OVX-induced groups of rats is shown in Fig. 7. The OVX-induced group rats had higher levels of cytokines, which were considerably (P < 0.001) reduced by bryodulcosigenin administration.

## Discussion

Osteoporosis after menopause is a condition that affects the bones, making them weaker and more prone to fractures. The exact cause of the disease is not fully understood, but it is believed to be linked to the decline in estrogen levels that occurs during menopause^25^. However, various investigations suggest that the aging, endocrine disorders, genetic factors, nutritional and calcium malabsorption play a significant role in the expansion of osteoporosis26. Bone metabolism is a continuous process in which old bone is removed (resorbed) by osteoclasts and new bone is formed (synthesized) by osteoblasts. In postmenopausal women, the decrease in estrogen levels can disrupt this balance and lead to an increase in osteoclast activity and a decrease in osteoblast activity. This results in a net loss of bone mass, making the bones more fragile and susceptible to fractures. This is the main reason behind the development of osteoporosis after menopause^26^^,^ 27.

Bilateral OVX is a commonly used animal model to study postmenopausal osteoporosis. In OVX, the ovaries of adult female rats are surgically removed, which leads to a rapid decline in estrogen levels and mimics the natural changes that occur during menopause in humans. This model is popular in research, because it is reliable, stable, repeatable, and has a high success rate in inducing bone loss similar to that seen in early postmenopausal women^3^. OVX-induced bone loss in rats is widely accepted as a reliable animal model for studying the pathophysiology and treatment of postmenopausal osteoporosis^28^.

OVX-induced osteoporosis is thought to occur due to a disruption of the balance between bone growth and resorption8 ^,^ 29. The loss of estrogen after OVX leads to an increase in oxidative stress, which is caused by an overproduction of reactive oxygen species (ROS). This increase in oxidative stress results in the production of cytokines, which in turn can stimulate the formation of osteoclasts (cells responsible for bone resorption) and inhibit the formation of osteoblasts (cells responsible for bone formation)^30^^,^ 31. This leads to a net loss of bone mass and the development of osteoporosis. The enhanced deposition of ROS after OVX also causes inflammation and increases the production of inflammatory cytokines that can further drive the bone loss and osteoclastogenesis^5^. Previous report suggests that the sufficient intake of vitamin D and calcium suppressed the osteoporosis incidence^5^. Cholecalciferol D3 is important for the absorption of phosphorous and calcium in the body, which are required for bone expansion. Phosphorous and calcium also play an important role as bone mineralization markers^8^^,^ 32. The level of mineral in OVX-infected rats was altered in this study, and bryodulcosigenin significantly restored the level of nutrient.

OVX is the mostly used animal method for the estimation of postmenopausal osteoporotic disease^13^. Hormones play significant role in the expansion and progression of osteoporosis. Estrogen deficiency is a major risk factor for osteoporosis, a condition characterized by low bone density and increased susceptibility to fractures^5^^,^ 33. Estrogen plays an important role in maintaining bone health by promoting the formation of bones and inhibiting the breakdown of the existing ones. When estrogen levels decline, as they do during menopause, the balance between bone formation and breakdown shifts in favor of breakdown, leading to decreased bone density and increased risk of fractures^34^^,^ 35. Additionally, low estrogen levels also can contribute to weight gain, which can further increase the risk of osteoporosis^24^.

Uterus is the significant target organ for the estrogen. In this work, we discovered that OVX-induced rats had increased uterus tissue weight, and that bryodulcosigenin treatment reduced uterus weight along with body weight. Previous research had found a similar outcome^24^. During the osteoporosis, the hormones level was suppressed due to bone loss and suppression of nutrients^36^^,^ 37. LH and FSH are two hormones produced by the pituitary gland that play a crucial role in the menstrual cycle and ovulation. FSH stimulates the growth and development of the ovarian follicles, which contain immature eggs^38^^,^ 39. LH, on the other hand, triggers ovulation by causing the dominant follicle to release its egg. E2 is a type of estrogen hormone that is produced by the developing ovarian follicles. As the follicles grow, they begin to produce increasing amounts of estradiol^40^. This increase in estradiol has a negative feedback effect on the hypothalamic-pituitary-ovarian axis, which regulates the menstrual cycle. Specifically, the rising levels of estradiol inhibit the release of FSH and LH through a feedback mechanism, thus preventing ovulation until the ovary has a dominant follicle that is ready to ovulate. Once the ovary is ready, the E2 level will drop and then the LH will surge, which triggers ovulation^24^. The hormone levels of OVX-induced group rats were disrupted, and bryodulcosigenin treatment significantly restored the hormone levels.

In osteoporosis, a chronic bone disorder characterized by reduced bone density and increased fracture risk, various inflammatory cytokines play crucial roles in the regulation of bone remodeling. Cytokines are among the key cytokines involved in the pathophysiology of osteoporosis. TNF-α is a pro-inflammatory cytokine that stimulates osteoclast formation and activity. It promotes bone resorption by enhancing the differentiation of osteoclast precursors and increasing their lifespan. Excessive TNF-α levels are associated with increased bone loss^41^^–^ 44. IL-1β is another pro-inflammatory cytokine that contributes to osteoclast activation and bone resorption. It stimulates the production of other inflammatory mediators, amplifying the inflammatory response in bone tissues. Elevated IL-1β levels are linked to bone loss in osteoporosis. IL-6 has dual effects on bone metabolism. While it can stimulate osteoclast activity, leading to bone resorption, it also plays a role in osteoclast inhibition through the regulation of OPG and RANKL expression. Elevated IL-6 levels are associated with bone loss in certain conditions. IFN-γ, typically associated with immune responses, can influence bone remodeling. It has been shown to enhance osteoclast activity indirectly by modulating the RANKL/OPG balance^43^^,^ 45. In certain contexts, IFN-γ may contribute to bone loss, especially in inflammatory conditions. The intricate interplay between these cytokines, along with other factors such as receptor activator of nuclear factor-kappa B (RANK) and OPG signaling, determines the balance between bone resorption and bone formation. In osteoporosis, an imbalance favoring bone resorption over formation results in net bone loss. Therapeutic strategies targeting these inflammatory cytokines, such as the use of biologics that inhibit TNF-α or IL-1β, are being explored as potential interventions to modulate bone turnover in osteoporosis.

TGF-β and IGF are important markers involved in the functioning and regulation of osteoblasts. TGF-β stimulates bone regeneration and is involved in the regulation of differentiation, immunological function, and expansion of osteoblasts. These growth factors play a crucial role in bone formation and repair, and their levels can be affected by various factors such as age, disease, and hormonal changes25. IGF is the significant metabolic hormones, involved in the regulating of bone homeostasis and balancing the normal bone growth25,46. Bryodulcosigenin treatment considerably revered the level of TGF-β and IGF level, showed the improvement of bone formation.

It is generally recognized that osteoporosis caused by estrogen insufficiency causes bone loss and slows bone metabolism25,47. Bone metabolism is a dynamic process that is kept in check by a dynamic balance between osteoclast-controlled bone resorption and osteoblast-controlled bone formation^25^. Various bone turnover indicators, such as osteoprotegerin, CTX, TRAP, and osteocalcin, were employed in the animal and clinical studies to examine the function of bone production and resorption^10^^,^ 48. OVX induced group rats exhibited the suppressed level of bone turnover markers, and bryodulcosigenin treatment considerably restored the bone turnover markers. A similar result was observed in previous research^25^.

It is well known that bone mineralization and remodeling is balanced by the action of estrogen, which acts via regulating homeostasis among osteoblast and osteoclast^9^. Bone formation markers like OC are synthesized via osteoblasts and they directly represent to their specific functions^5^. The increasing level of bone formation in menopausal women indicates osteoporosis, and a similar result was found in this study, in which OVX showed an elevated level of OC, which could be attributed to osteoblast development to compensate for bone loss caused by estrogen shortage.

RANKL/OPG axis pathway plays a critical role in maintaining the balance between bone resorption and formation. RANKL is a protein that stimulates osteoclasts, the cells responsible for bone resorption, while OPG is a protein that acts as a decoy receptor for RANKL and blocks its activity, thus inhibiting osteoclast formation. The balance between RANKL and OPG determines the level of osteoclast activity, and thus the rate of bone resorption. Disruption of this balance can lead to bone disorders such as osteoporosis, which is characterized by a decrease in bone mass and increased risk of fractures^5^^,^ 11. They play a crucial factor in mediating the osteoclasts differentiation. OPG generated via lineage of osteoblasts has a suppressive effect on the bone formation, whereas increased RANKL is mainly related to bone fractures, consequently resultant enhanced the formation of osteoclasts^5^. We observed the reduction level of OPG and boosted level of RANKL in the OVX induced group rats. Bryodulcosigenin treatment remarkably restored the level of OPG and RANKL and suggested the protection of the rats against the OVX induced bone loss in the rats via improved the OPG/RANKL ratio. The study supports that bryodulcosigenin could be protective against the bone loss during the postmenopausal women.

## Conclusion

In this experimental study, results showed that the bryodulcosigenin considerably suppressed the body weight and enhanced the uterine weight. Bryodulcosigenin considerably suppressed the BMD in the whole femur, caput femoris, distal femur, and proximal femur. Bryodulcosigenin remarkably suppressed the levels of bALP, TRAP, and CTX and boosted the levels of osteocalcin, osteoprotegerin, and OT. It also suppressed the level of FSH, and LH and boosted the level of E2. Bryodulcosigenin considerably boosted the levels of TGF-β, and IGF and reduced the levels of TNF-α, IFN-γ, IL-6, and IL-1β. Additionally, more mechanistic action of bryodulcosigenin needs to be scrutinized the osteoporosis effect.

## Data Availability

The data will be available upon request.

## References

[1] Xu H, Liu T, Hu L, Li J, Gan C, Xu J, Chen F, Xiang Z, Wang X, Sheng J (2019). Effect of caffeine on ovariectomy-induced osteoporosis in rats. Biomed Pharmacother.

[2] Zhang Z, Zhao Q, Liu T, Zhao H, Wang R, Li H, Zhang Y, Shan L, He B, Wang X, Huang L, Hao D, Sun H (2020). Effect of Vicenin-2 on ovariectomy-induced osteoporosis in rats. Biomed Pharmacother.

[3] Moghazy H, Mahmoud AA, Elbadre H, Aziz HO (2020). Protective Effect of Oxytocin Against Bone Loss in a Female Rat Model of Osteoporosis. Reports Biochem Mol Biol.

[4] Pisani P, Renna MD, Conversano F, Casciaro E, Di Paola M, Quarta E (2016). Major osteoporotic fragility fractures: Risk factor updates and societal impact. World J Orthop.

[5] Yang T, Wang Q, Qu Y, Feng C, Li C, Yang Y (2021). Protective effect of rhaponticin on ovariectomy-induced osteoporosis in rats. J Biochem Mol Toxicol.

[6] Xie CL, Park KH, Kang SS, Cho KM, Lee DH (2021). Isoflavone-enriched soybean leaves attenuate ovariectomy-induced osteoporosis in rats by anti-inflammatory activity. J Sci Food Agric.

[7] Wang QL, Huo XC, Wang JH, Wang DP, Zhu QL, Liu B (2017). Rutin prevents the ovariectomy-induced osteoporosis in rats. Eur Rev Med Pharmacol Sci.

[8] Liu T, Xiang Z, Chen F, Yin D, Huang Y, Xu J (2018). Theabrownin suppresses in vitro osteoclastogenesis and prevents bone loss in ovariectomized rats. Biomed Pharmacother.

[9] Srikanta P, Nagarajappa SH, Viswanatha GL, Handral M, Subbanna R, Srinath R (2011). Name of the article. J Chinese Integr Med.

[10] Roch PJ, Henkies D, Carstens JC, Krischek C, Lehmann W, Komrakova M (2020). Ostarine and Ligandrol Improve Muscle Tissue in an Ovariectomized Rat Model. Front Endocrinol (Lausanne).

[11] Wolski H, Drews K, Bogacz A, Kaminski A, Barlik M, Bartkowiak-Wieczorek J (2016). The RANKL/RANK/OPG signal trail: Significance of genetic polymorphisms in the etiology of postmenopausal osteoporosis. Ginekol Pol.

[12] Liu W, Xie G, Yuan G, Xie D, Lian Z, Lin Z, Ye J, Zhou W, Zhou W, Li H, Wang X, Feng H, Liu Y, Wang X, Feng Y, Liu H, Wang X, Front Pharmacol (2021). 6′-O-Galloylpaeoniflorin Attenuates Osteoclasto-genesis and Relieves Ovariectomy-Induced Osteoporosis by Inhibiting Reactive Oxygen Species and MAPKs/c-Fos/NFATc1 Signaling Pathway. Front Pharmacol.

[13] Li M, Hao L, Li L, Liu L, Chen G, Jiang W, Xu W, Zhu C, Yao G, Fang S (2020). Cinnamtannin B-1 Prevents Ovariectomy-Induced Osteoporosis via Attenuating Osteoclastogenesis and ROS Generation. Front Pharmacol.

[14] Jyotsna V (2013). Postmenopausal hormonal therapy: Current status. Indian J Endocrinol Metab.

[15] Lewiecki EM (2017). Osteoporosis: Treat-to-Target. Curr Osteoporos Rep.

[16] Lewiecki EM, Cummings SR, Cosman F (2013). Treat-to-target for osteoporosis: Is now the time?. J Clin Endocrinol Metab.

[17] Senthilkumar K, Venkatesan J, Kim SK (2014). Marine derived natural products for osteoporosis. Biomed Prev Nutr.

[18] Şöhretoğlu D, Renda G (2020). Medicinal natural products in osteoporosis. Ann Rep Med Chemistry.

[19] Li R, Chen C, Liu B, Shi W, Shimizu K, Zhang C (2022). Bryodulcosigenin a natural cucurbitane-type triterpenoid attenuates dextran sulfate sodium (DSS)-induced colitis in mice. Phytomedicine.

[20] Ukiya M, Akihisa T, Yasukawa K, Tokuda H, Toriumi M, Koike K (2002). Anti-inflammatory and anti-tumor-promoting effects of cucurbitane glycosides from the roots of Bryonia dioica. J Nat Prod.

[21] Nakano K, Kanai Y, Murakami K, Takaishi Y (1995). Cucurbitacin glycosides from Cabeça-de-negro. Phytochemistry.

[22] Oobayashi K, Yoshikawa K, Arihara S (1992). Structural revision of bryonoside and structure elucidation of minor saponins from Bryonia dioica. Phytochemistry.

[23] Repo MA, Bockman RS, Betts F, Boskey AL, Alcock NW, Warrell RP (1988). Effect of gallium on bone mineral properties. Calcif Tissue Int.

[24] Zhang D-W, Wang Z-L, Qi W, Zhao G-Y (2014). The effects of Cordyceps sinensis phytoestrogen on estrogen deficiency-induced osteoporosis in Ovariectomized rats. BMC Complement Altern Med.

[25] Liu H, Zhang H, Fan H, Tang S, Weng J (2020). The preventive effect of Cuscutae Semen polysaccharide on bone loss in the ovariectomized rat model. Biomed Pharmacother.

[26] Saedi A, Stupka N, Duque G, Michel MC (2020). Handbook of Experimental Pharmacology.

[27] Orimo H (2003). Pathogenesis of osteoporosis--overview. Nippon Rinsho.

[28] Li CW, Liang B, Shi XL, Wang H (2015). Opg/Rankl mRNA dynamic expression in the bone tissue of ovariectomized rats with osteoporosis. Genet Mol Res.

[29] Zhang D, Zhang S, Jiang K, Li T, Yan C (2020). Bioassay-guided isolation and evaluation of anti-osteoporotic polysaccharides from Morinda officinalis. J Ethnopharmacol.

[30] Putri CA, IGA Adnyana IK (2016). Preventive effect of Peperomia pellucida (L.) Kunth herbs on ovariectomy-induced osteoporotic rats. J Chinese Pharm Sci.

[31] Zhang J, Zong L, Bai D (2019). Boeravinone b promotes fracture healing in ovariectomy-induced osteoporotic rats via the regulation of NF-κB p65/IκB-α/SIRT-1 signaling pathway. Trop J Pharm Res.

[32] Dong XL, Yu WX, Li CM, Zhou LP, Wong MS (2020). Chuanxiong (Rhizome of Ligusticum chuanxiong) Protects Ovariectomized Hyperlipidemic Rats from Bone Loss. Am J Chin Med.

[33] YM Lee, Kim IS, Lim BO (2019). Black Rice (Oryza sativa L.) Fermented with Lactobacillus casei Attenuates Osteoclastogenesis and Ovariectomy-Induced Osteoporosis. Biomed Res Int.

[34] Saleh NK, Saleh HA (2011). Olive Oil effectively mitigates ovariectomy-induced osteoporosis in rats. BMC Complement Altern Med.

[35] Li S, Chen R, Luo K, Guo Y, Xiao M, Du G (2017). Areca nut extract protects against ovariectomy-induced osteoporosis in mice. Exp Ther Med.

[36] Jia L, Tu Y, Jia X, Du Q, Zheng X, Yuan Q, Zheng L, Zhou X, Xu X (2021). Probiotics ameliorate alveolar bone loss by regulating gut microbiota. Cell Prolif.

[37] Wu K, Gong Z, Zou L, Ye H, Wang C, Liu Y (2019). Sargassum integerrimum inhibits oestrogen deficiency and hyperlipidaemia-induced bone loss by upregulating nuclear factor (erythroid-derived 2)-like 2 in female rats. J Orthop Transl.

[38] Wang J, Zhang W, Yu C, Zhang X, Zhang H, Guan Q, Zhao J, Xu J, Guan J (2015). Follicle-stimulating hormone increases the risk of postmenopausal osteoporosis by stimulating osteoclast differentiation. PLoS One.

[39] Chin KY (2018). The relationship between follicle-stimulating hormone and bone health: Alternative explanation for bone loss beyond oestrogen. Int J Med Sci.

[40] Zhang Y, Liu MW, He Y, Deng N, Chen Y, Huang J (2020). Protective effect of resveratrol on estrogen deficiency-induced osteoporosis though attenuating NADPH oxidase 4/nuclear factor kappa B pathway by increasing miR-92b-3p expression. Int J Immunopathol Pharmacol.

[41] Condi FLF, Soares JM, Teodoro WR, Veloso AP, Parra ER, Jesus Simoes, MD Baracat (2012). The effects of conjugated estrogen, raloxifene and soy extract on collagen in rat bones. Climacteric.

[42] Carbonel AAF, Vieira MC, Simões RS, Lima PDA, Fuchs LFP, Girão ERC, Cicivizzo GP, Sasso GRS, de Moraes LPC, Soares JM, Baracat EC, Simões MJBC, Simões MJBC, Simões MJBC, Simões MJBC, Simões MJBC, Simões MJBC (2020). Isoflavones improve collagen I and glycosaminoglycans and prevent bone loss in type 1 diabetic rats. Climacteric.

[43] McLean RR (2009). Proinflammatory cytokines and osteoporosis. Curr Osteoporos Rep.

[44] Yang DH, Yang MY (2019). The Role of Macrophage in the Pathogenesis of Osteoporosis. Int J Mol Sci.

[45] Ginaldi L, Di Benedetto MC, De Martinis M (2005). Osteoporosis, inflammation and ageing. Immun Ageing.

[46] Giustina A, Mazziotti G, Canalis E (2008). Growth hormone, insulin-like growth factors, and the skeleton. Endocr Rev.

[47] Liu H, Xiong Y, Wang H, Yang L, Wang C, Liu X, Wu Z, Li X, Ou L, Zhang R, Zhu Z (2018). Effects of water extract from epimedium on neuropeptide signaling in an ovariectomized osteoporosis rat model. J Ethnopharmacol.

[48] Shiraishi A, Takeda S, Masaki T, Higuchi Y, Uchiyama Y, Kubodera N, Sato K, Ikeda K, Nakamura T, Matsumoto T, Ogata E (2000). Alfacalcidol inhibits bone resorption and stimulates formation in an ovariectomized rat model of osteoporosis: Distinct actions from estrogen. J Bone Miner Res.

